# Modeling oil price uncertainty effects on economic growth in Mexico: a sector-level analysis

**DOI:** 10.1007/s11356-022-20711-2

**Published:** 2022-05-28

**Authors:** Domingo Rodríguez-Benavides, Roldán Andrés-Rosales, María de la Cruz del Río-Rama, Muhammad Irfan

**Affiliations:** 1 Department of Applied Econometrics, Metropolitan Autonomous University, 02200 Mexico City, State of Mexico Mexico; 2 Department of Social Sciences, Faculty of Higher Studies Cuautitlan-UNAM, 54714 Mexico, State of Mexico Mexico; 3 Universidade de Vigo, Facultade de Ciencias Empresariais e Turismo, 32004 Ourense, España; 4 School of Management and Economics, Beijing Institute of Technology, Beijing, 100081 China; 5 Center for Energy and Environmental Policy Research, Beijing Institute of Technology, Beijing, 100081 China; 6 Department of Business Administration, ILMA University, Karachi, 75190 Pakistan

**Keywords:** Oil price volatility, Economic sectors, Multivariate VAR-GARCH in mean, Asymmetric effects, Economic growth in Mexico

## Abstract

This paper analyzes the impact of international oil price uncertainty on the different economic sectors (primary, secondary, and tertiary) in Mexico in the period 1993:1–2020:4 through a bivariate structural vector autoregressive (VAR) model with a generalized autoregressive conditional heteroskedasticity (GARCH) in mean to capture the impact of oil volatility on economic growth at the sectoral level of economic activity. The results show that the uncertainty of the international price of oil has a differentiated effect on the different sectors of economic activity in Mexico since it does not influence the primary sector; it negatively impacts the secondary sector, and there is mixed evidence in the tertiary sector. Additionally, evidence is provided that both positive and negative shocks to the international oil price have asymmetric effects at the sectoral level in Mexico. The results highlight the need to implement public policies, at the country level, that help mitigate the effect of uncertainty in the oil market and promote economic stability at the sector level.

## Introduction

The Mexican economy is currently considered as one of the main emerging economies (IMF 2021) by presenting low per capita income and high economic growth (it is in the process of industrialization, and the foreign trade sector is on the rise). It is estimated that emerging economies as a group could double the size of those countries in the Group of Seven (G-7) by the year 2040 (Hawksworth et al. 2017). Given the possible shift of economic power to emerging market economies in the coming decades, it is relevant to examine whether these economies are vulnerable to external shocks, which they cannot control by definition and that may have adverse effects on their real economic activity (Azad and Serletis 2021).

The confinement caused by the SARS-Cov-2 health crisis (Yang et al. 2021; Jinru et al. 2021; Wen et al. 2022) and, consequently, the notable economic slowdown that was triggered worldwide (Chandio et al. 2021; Elavarasan et al. 2021; Irfan et al. 2022), as well as the recent armed conflict in Ukraine show that the world economy remains highly vulnerable and dependent on fluctuations in the price of oil and other primary energy sources. In the first case, it was shown that the price of oil fell to unsuspected levels, even becoming negative, while in the second, it has contributed to fueling the inflationary pressures already generated by the scarcity and lack of supply caused by the COVID-19 pandemic (Ahmad et al. 2022; Huang et al. 2022; Razzaq et al. 2020, 2022). Hence, the need to quantify the impact of fluctuations in oil prices and their volatility on real activity, not only for developed economies but also for emerging countries.

There is an extensive empirical literature that investigates whether positive oil price shocks cause recessions in advanced countries (Khan et al. 2021; Sun et al. 2021a; Razzaq et al. 2021), such as the USA (Edelstein and Lutz 2009; Elder and Serletis 2009; Kilian and Vigfusson 2011b). Comparably, the literature on the Mexican economy is quite scarce. Given the importance of oil in the world economy, the economic effect of its price volatility has received considerable attention in the last decades (Van Eyden et al. 2019). The interest is justified by the fact that oil is not only the most commercialized commodity in the world but also remains as the main source of energy used worldwide (World Oil Outlook 2021). In addition, the price of oil, unlike that of other commodities, has been more volatile and has had a greater degree of uncertainty (Wachtmeister et al. 2018).

Oil price shocks affect real economic activity through different supply and demand channels (Anuar et al. 2021; Sinha et al. 2022; Żywiołek et al. 2022). It has been argued that seven of the eight recessions during the postwar period were caused by an increase in oil prices (Hamilton 1983). Researchers have shown an increased interest in the empirical relationship between the price of oil and macroeconomic activity since the oil crisis of the 1970s and the subsequent stagflation. Even though there is a wealth of evidence on the negative effect of higher oil prices on aggregate economic activity, not enough attention has been paid to the impact of oil price shocks on the productive sectors of Mexico. In spite of the large amount of empirical work that has documented the negative effect of international oil price uncertainty on some macroeconomic variables such as aggregate product, investment, and manufacturing for different countries and even for some stock market indicators, not enough attention has been paid to the impact that such uncertainty may have at the sectoral level and even less for an economy like the Mexican. The literature on the relationship between oil price shocks and fluctuations in economic activity has approached the issue from different angles, awarding great importance to the asymmetric impacts that oil shocks can have on the economy (Kandemir Kocaaslan 2020). Positive shocks are considered to inhibit economic growth more than negative shocks tend to stimulate it.

Adjustment costs have also been offered as an explanation for asymmetric effects (Hamilton 1985), in the sense that an increase in oil prices not only prevents production growth directly but also reduces production indirectly through these costs. However, a fall in oil prices could also have negative effects related to adjustment costs, though some positive economic effects are likely to outweigh each other. The asymmetry has also been attributed to the transmission between the price of crude oil and that of petroleum products, as the prices of some of these products react more quickly to oil prices compared to lower oil prices (Huntington 1998; Brown and Yücel 2002).

Other authors, such as Davis (1987a, b) and Davis and Haltiwanger (2001), indicate that sectoral reallocation also produces an asymmetry in the response of production growth to oil price shocks, where oil price shocks lead to the reallocation of capital and labor from the affected to the benefited sectors. This reallocation of specialized factors of production between sectors is costly and worsens the adverse effect of the rise (fall) in oil prices and diminishes the favorable effect of the fall (rise) in oil prices for countries that import (export) oil. On his part, Hamilton (1985) developed a model for an economy in which the reallocation of specialized factors of production between sectors is rather costly. In this model, relative price shocks increase general unemployment as workers in depressed sectors decide to wait for recovery instead of moving to sectors that enjoy better conditions.

In the study of the macroeconomic effects coming from oil price uncertainty, the theory of real options or the theory of investment under uncertainty is common ground. This theory posits that companies are likely to delay irreversible investment decisions when there is uncertainty about the price of oil, especially when the cash flow of investments depends on the price of oil (Bernanke 1983; Pindyck 1991; Elder and Serletis 2009; Balashova and Serletis 2021). In this context, oil price uncertainty, defined as an unforeseen change in the future price, influences the company’s expectations about current production and investment decisions. It is worth noting that uncertainty is different from volatility, according to Grier and Perry (1998), since it is possible to consider uncertainty as unpredictable fluctuations, while variability captures predictable and unpredictable fluctuations.

A large amount of the literature has focused on determining whether the uncertainty or volatility of international oil prices impact economic activity at the aggregate level; however, not enough interest has been placed on the effects at the sectoral level (Bibi et al., 2021). This paper will investigate the impact of international oil price uncertainty on the economic growth of the primary, secondary, and tertiary sectors of the economy in Mexico during the period 1993–2020 on a quarterly basis. The objective is to answer the following questions: Does the economic growth of different sectors of economic activity in Mexico respond in the same way to international oil price uncertainty? And, is there evidence of asymmetric effects of international oil price shocks at the sectoral level in Mexico? The main hypothesis is that, given the heterogeneous levels of capital intensity in the diverse processes carried out by the sectors of economic activity in Mexico, international oil price uncertainty causes a differentiated effect at the sector level. This is demonstrated through the bivariate VAR-GARCH in the mean model, in which price uncertainty is allowed to impact the economic growth of each sector, but the variance of such economic growth is not allowed to affect the growth of international oil prices, an assumption that is fully justified in the case of a relatively small oil-importing economy in recent decades. The quarterly average of the refiner acquisition cost of crude oil (RAC), prepared by the Department of Energy, serves as a proxy for the international price of oil, while information on the sector’s GDP comes from the Economic Information Bank (BIE) of the National Institute of Statistics and Geography (INEGI).

In what follows, the “Review of the empirical literature” section will present a brief review of the empirical literature; the “Econometric methodology” section will explain the econometric methodology; “The data” section will show the empirical results, and finally, the “Empirical evidence of uncertainty in the productive sectors” section will state the main conclusions.

## Review of the empirical literature

Studies on the impact of oil price shocks on macroeconomic activities can be classified into three large groups: those that examine the impact of oil price shocks, those that incorporate the asymmetric nature of oil price shocks, and studies that address the impact of oil price uncertainty (Ahmed et al. 2012).

Some economists argue that oil price shocks are perhaps more important than currency shocks as a determinant of postwar US recessions (Kilian and Vigfusson 2011a). Hamilton (1983) is a pioneer within the first group of studies, showing that, with the exception of one, all the recessions that occurred in the USA between the end of World War II and 1973 were preceded by a strong increase in the price of oil. Cunado and Perez de Gracia (2005) also find that oil price shocks have a significant effect on economic growth for a sample of European countries. In a more recent study, Cunado et al. (2015) analyze the macroeconomic impact of oil shocks in four of the largest oil-consuming economies in Asia through a structural autoregressive vector, where sign restrictions help identify the three types of shocks analyzed—of oil supply, specifics of oil demand, and of oil demand driven by world economic activity. Their results suggest that economic activity and prices respond differently to these three types of oil price shocks.

Studies that use standard VAR models to analyze the relationship between the price of oil and the economy do not usually take into account the volatility inherent to the price of oil. These studies implicitly assume that history always repeats itself (Aye et al. 2014). However, it has been shown that the evolution of oil prices has displayed different volatilities at different intervals of time, as well as variable volatility in its evolution over time (Orhan and Köksal 2012).

The second group of studies mentioned focuses on the asymmetric impact of oil price crises on macroeconomic activities (Mork 1989; Davis 1987a; Lee et al. 1995; Davis and Haltiwanger 2001; Cunado and Perez de Garcia 2005). Under the symmetry assumption, an increase in the price of oil reduces the level of aggregate production, while a fall in the price of oil increases economic activity. However, some have pointed out that this symmetry is apparently inconsistent with the slow growth observed in the mid-1980s, which was accompanied by a large drop in oil prices. Mork et al. (1994) showed that, while an increase in the price of oil has a negative and significant impact on production growth in the USA, a fall in the price of oil does not lead to higher growth. Mork et al. (1994) argue that this asymmetry is also present in most other OECD countries. Huang et al. (2005) found asymmetric effects of oil price shocks on economic growth as well in the USA, Canada, and Japan. Despite said evidence of asymmetric effects, there is no consensus on the level and importance of the asymmetry that prevails in the effects of an oil price crisis on macroeconomic activities (Hooker 1996; Mork 1989; Lee et al. 1995).

The third group of studies has focused on investigating the impact of oil price uncertainty on production through a bivariate GARCH in mean model, estimating the asymmetric response of economic activity to positive and negative oil price shocks. Elder and Serletis (2010) investigated the relationship between oil price uncertainty and US investment. Aye et al. (2014) did the same for South African manufacturing production. Maghyereh et al. (2019) observed that higher oil price uncertainty had an adverse effect on industrial production in Turkey and Jordan. Rafiq and Salim (2014) studied the impact of oil price volatility in six large emerging Asian economies, using time series and cross-section econometric techniques.

Cheng et al. (2019) investigated the dynamic impacts of oil price uncertainty on the Chinese economy. Rodríguez and López (2019) analyzed the impact of international oil price uncertainty on aggregate production and investment in Mexico. Jo (2014) studied the effect of oil price uncertainty on global real economic activity. Ahmed and Wadud (2011) examined the impact of oil price uncertainty on Malaysia’s macroeconomic activities and its monetary policy response.

Ahmed et al. (2012) examine the impact of uncertainty in oil prices on US industrial production. Punzi (2019) evaluated the macroeconomic implications of energy price volatility using a dynamic stochastic general equilibrium (DSGE) model in 10 Asian economies. Maghyereh and Abdoh (2020) investigated how uncertainty about oil prices affects investment in the USA. Finally, Elder (2018) examined the effect of oil price volatility on disaggregated measures of industrial production, which are the special aggregates by market groups computed by the Federal Reserve Board.

To sum up, most of these studies find that international oil price uncertainty has adverse effects on a number of variables, such as economic growth, private and public investment, and productive sectors, and that the impacts of positive and negative shocks are asymmetrical.

Recently, there has been a growing interest in the study of the interaction between some pollutants, clean energy, and some processes such as globalization, among others, in economic activity. Several studies employ time series techniques to examine the benefit of renewable energy in environmental mitigation. For example, for the MENA (Middle East and North Africa) countries, Sun et al. (2022) find that it is possible to reduce carbon dioxide emissions if biomass energy is increased in production processes. Sohail et al. (2022) show evidence that political stability increases clean energy consumption in Pakistan and contributes to improving environmental quality in the short term. Similarly, Sun et al. (2021b) examine the relationship between carbon dioxide emissions and technical advances (investment in research and development) and find that technological innovations had a negative impact on carbon dioxide emissions in the USA between 1980 and 2018. Sohail et al. (2022) explore the asymmetric effects of political instability on clean energy consumption and CO_2_ emissions. Their results from the ARDL model show that political stability decreases damage, while with the nonlinear approach, they find that political instability not only reduces clean energy consumption but also damages environmental quality. Li et al. (2022), through nonlinear panel modeling techniques, find that the development of the insurance sector has asymmetric effects on CO_2_ emissions, specifically, a positive shock in the development of the insurance sector increases CO_2_ and a negative shock in the development of the insurance sector decreases CO_2_ in the long term in highly polluting economies. Chien et al. (2021) provide evidence that technological innovation and renewable energies are inversely related to environmental degradation and that renewable energies contribute to reducing emissions in the short term. Chien et al. (2022) show that solar power is negatively and significantly associated with long-term CO_2_ emissions and that eco-innovation has proven to be the most important channel to mitigate CO_2_ emissions in China.

## Econometric methodology

The empirical model used is based on the proposal by Elder and Serletis (2010), which consists of estimating a bivariate structural vector autoregressive (SVAR) with multivariate generalized autoregressive conditional heteroskedasticity in the mean (MGARCH-M) model. The main characteristic of this model is that it imposes the restriction that the conditional variance of economic growth in the growth rate of the international real price of oil is equal to zero, thus allowing the variance of the real international price of oil to have an effect on economic growth. The main advantage of estimating all the parameters simultaneously is that it generates internally consistent estimates, which avoids the problem of the “generated regressor” (Maghyereh et al. 2017). The structural VAR for the conditional mean is given as follows:

1 $$B{y}_t=C+{\Gamma}_1{y}_{t-1}+{\Gamma}_2{y}_{t-2}+\dots +{\Gamma}_{\mathrm{p}}{y}_{t-p}+\Lambda (L){H}_t^{\frac{1}{2}}+{\varepsilon}_t$$

where *y*_*t*_ is a vector of endogenous variables over time *t*, in this case, the annualized quarterly growth rates of the real GDP of each sector and of the real international price of oil. *B* is a square matrix with a 2*x*2 dimension *ϵ*_*t*_ ∣ *ψ*_*t* − 1_ : *iid N*(0, *H*_*t*_), while ${H}_t^{1/2}$ is a diagonal matrix, Λ(*L*) is a polynomial matrix with a lag operator, *ψ*_*t* − 1_ is the set of information in time *t* − 1, *p* is the length of the lags, and *T* is the sample size. The term $\Lambda (L){H}_t^{1/2}$ captures the impact of international real oil price uncertainty on economic growth at the sector level. The orthogonalized structural innovations (*F*_*t* − 1_) in the model are related to the choice of the *N*(*N* − 1)/*N* free parameters in matrix *B*; they are assumed to be normally and independently distributed. Furthermore, structural shocks *ε*_*t*_ are not assumed to be conditionally correlated.

In order to ensure the identification of structural responses, a necessary and sufficient number of identification restrictions are imposed on matrix *B* (Maghyereh et al. 2017). The usual way to accomplish this task is to impose zero constraints through the Cholesky decomposition, analogous to a conventional structural VAR model. Following the identification procedure used by Elder and Serletis (2010), Bredin et al. (2011), and Maghyereh et al. (2017), we restrict matrix *B* so that sectoral economic growth responds instantly to innovations in real oil price growth, but not the other way around. Hence, one parameter is allowed to remain free in the matrix in the bivariate VAR.

In the above specification, oil price volatility is measured by the conditional standard deviation of structural innovations ${H}_t^{1/2}$. In this sense, ${H}_t^{1/2}$ is a measure of the conditional standard deviation of one quarter ahead of oil’s real price, which enables measuring the impact of oil price uncertainty shocks on the conditional mean of *y*_*t*_. The influence of oil price volatility on sectoral economic growth is measured through coefficient matrix Λ(*L*). Specifically, a significant negative element in Λ implies that oil price volatility tends to negatively affect the economic growth of the sector in question (Maghyereh et al. 2017). This term also captures any potential asymmetry in the effects on sectoral economic growth attributable to oil price shocks. If Λ(*L*) is negative, then the unforeseen positive and negative oil price shocks increase uncertainty in the oil market and, consequently, depress the economic growth of the sector in question in the short term (Elder and Serletis 2009, 2010).

Conditional variance *H*_*t*_ is specified as a bivariate GARCH, proposed by Engle and Kroner (1995), which can be written as

2 $${h}_t={C}_v+\sum_{i=1}^q{F}_i{\eta}_{t-i}+\sum_{j=1}^r{G}_j{h}_{t-j}$$

where ${\eta}_{t-i}= vec\left({\varepsilon}_{t-i}^{\prime }{\varepsilon}_{t-i}\right)$, *h* = *vec*(*H*_*t*_), ${\varepsilon}_t:{H}_t^{1/2}{z}_t$, *z*_*t*_ : *N*(0, *I*), *F*_*i*_ and *G*_*j*_ are *NxN* matrices, with *C*_*v*_ as an upper triangular matrix to make sure that *H*_*t*_ is defined as positive.

The proposal of Elder (2004), as well as Elder and Serletis (2010), suggests a modified version of this model to reduce the number of parameters in the variance function, which consists of assuming null contemporary correlations in structural disturbances, implying that *H*_*t*_ is a diagonal matrix. It is then possible to write the structural variance function as

3 $$\mathit{\operatorname{diag}}\left({H}_t\right)={C}_v+\sum_{i=1}^q{F}_i\mathit{\operatorname{diag}}\left({\eta}_{t-i}\right)+\sum_{j=1}^r{G}_j\mathit{\operatorname{diag}}\left({h}_{t-j}\right)$$

Equation (3) establishes that the conditional variance is a function of *q* lagged squared errors and of *r* lags of the same conditional variance, so that the matrices *F*_*i*_ and *G*_*i*_ are also diagonals (Maghyereh et al. 2017). The bivariate VAR model with GARCH in mean terms, given by Eqs. (1) and (3), are estimated simultaneously using the maximum likelihood method, as described by Elder (2004). The procedure involves maximizing the logarithm of the following likelihood function:

4 $$\mathit{\log}{L}_t\left(\theta \right)=\sum_{t=1}^T{l}_t\theta$$

described by Elder (2004), where *L*_*T*_ is the sample likelihood function and *θ* = (*B*, *C*, *A*_1_, *A*_2_, …*A*_*p*_, Λ, *F*, *G*) is a vector of structural parameters.

5 $${l}_t\theta =-\left(\frac{N}{2}\right)\log \left(2\pi \right)+\left(\frac{1}{2}\right)\log {\left|B\right|}^2-\left(\frac{1}{2}\right)\log \mid {H}_t\mid -\left(\frac{1}{2}\right)\log \left({\varepsilon}_t^{\prime }{H}_t^{-1}{\varepsilon}_t\right)$$

To obtain maximum likelihood estimates, Eq. (4) is numerically maximized with respect to the structural parameters using the method (BFGS). The multivariate GARCH of the VAR in the mean model enables analyzing the dynamic effects of the shock in one system variable on the conditional forecast of another variable through the impulse-response functions, which are capable of capturing the potentially asymmetric effects of oil price shocks (Elder and Serletis 2009, 2010; Maghyereh et al. 2017).

## The data

The data used here is the quarterly Gross Domestic Product (GDP) at constant 2013 prices in local currency for the primary, secondary, and tertiary sectors for the period that runs from the first quarter of 1993 to the fourth quarter of 2020, computed by the Economic Information Bank (BIE) of the National Institute of Statistics and Geography (INEGI) of Mexico. The international price of oil is approximated by the quarterly average of the composite refiner acquisition cost of crude oil (RAC), prepared by the Mexican Department of Energy. This price index is a weighted average of the costs of domestic and imported crude; it includes transportation costs and other fees paid by refineries, for which it reflects well the price of crude oil as an input for production (Elder and Serletis 2010), and it measures oil prices more broadly than other oil price indicators. This variable was included in the model both in constant dollars, deflated using the implicit price index of the United States GDP, and in constant pesos, using the exchange rate and the implicit price deflator of Mexico's GDP.

**Fig. 1:**
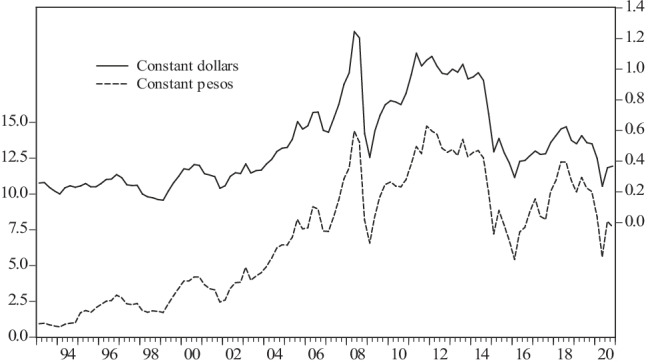
Evolution of international oil prices in real terms in dollars and pesos, 1993:1–2020:4. Source: authors using data from INEGI

The annualized growth rates of these variables were then computed. The evolution of the real international price of oil (RAC) both in dollars and in pesos is shown in Fig. 1. Oil prices in dollars and in pesos followed a similar course; however, from 1993 to 2006, the real international price of oil in pesos grew at a higher rate than this same price in dollars, but since 2006, both prices have an analogous behavior.

**Fig. 2:**
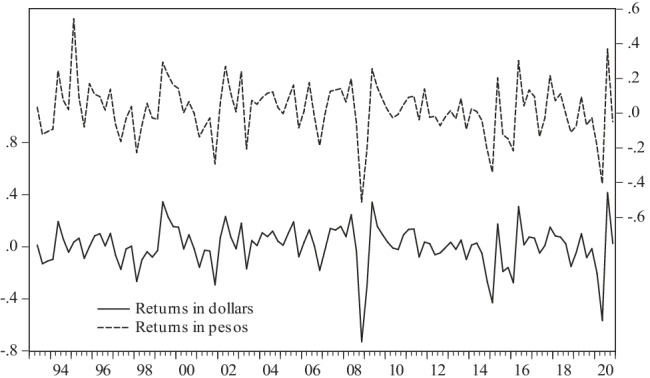
International oil price returns in dollars and in pesos, 1993:1–2020:4. Source: authors using data from INEGI

Figure 2 shows the evolution of returns to the real international price of oil, both in dollars and in pesos, where the scale in pesos is on the right axis, though only for illustrative purposes since both lines show practically the same trail.

**Fig. 3:**
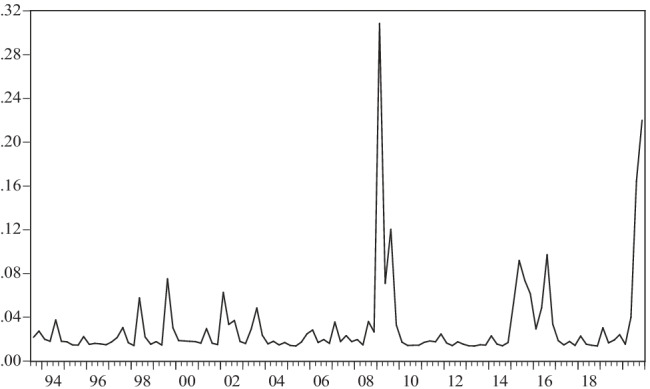
Conditional variance of returns to real oil prices in dollars according to an AR(1)-GARCH(1,1) model, 1993:2–2020:4. Source: authors using data from Energy Information Administration (EIA)

**Fig. 4:**
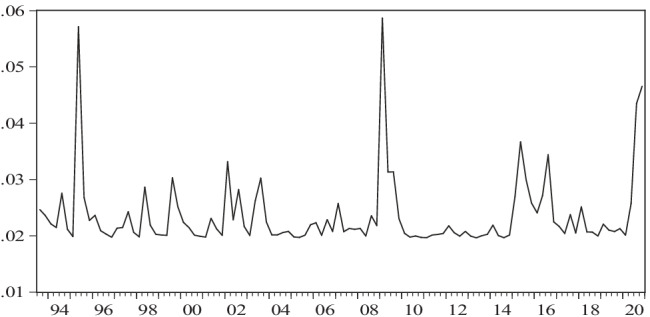
Conditional variance of returns to real oil prices in pesos according to an AR(1)-GARCH(1,1) model, 1993:2–2020:4. Source: authors using data from EIA and Banxico

The conditional variance will show the evolution of international oil price volatility, estimated through an AR(1)-GARCH(1,1) model for real prices in dollars and in pesos during the period of study. Figures 3 and 4 show that the estimated conditional variances behaved in a more or less similar way between 1993 and 2020, with the noteworthy exception that the variance in pesos is much greater than that estimated in dollars in the first years of the sample.

**Fig. 5:**
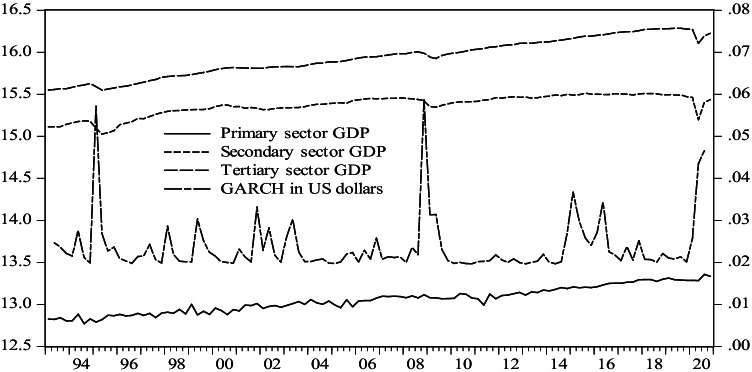
Conditional variance of returns to real oil prices in pesos according to an AR(1)-GARCH(1,1) model and GDP of the primary, secondary, and tertiary sectors in Mexico, 1993:2–2020:4. Source: authors using data from INEGI

Figure 5 presents the international oil price variances in dollars, estimated by the aforementioned AR(1)-GARCH(1,1) model, along with the GDP of the three economic sectors. The increase in the volatility of international oil prices appears to be more associated with the falls in economic activity in the secondary and tertiary sectors and less with the performance of the primary sector, especially during economic crises, such as in the years 1994–1995, 2008, and since 2020 due to COVID-19; this is even more noticeable when observing the estimated volatility for real oil prices calculated in pesos.

During the study period in Mexico, various events of economic and social significance were recorded. Different economic reforms were carried out; possibly, the main ones were embodied by the entry into force of the North American Free Trade Agreement (NAFTA) in early 1994, which was followed by the currency crisis at the end of the same year, also known as the Tequila effect due to its international repercussions. The next main event could be pinned to the international financial crisis of 2008. The crisis had a negative impact on the Mexican economy through exports, affecting industrial and manufacturing production.

More recently, like virtually every economy in the world, Mexico suffered the economic crisis caused by the coronavirus disease (COVID-19), largely brought about by quarantines ordered by governments around the world. The enormous losses caused by the pandemic and the simultaneous collapse of the oil price were a major shock to emerging markets. In the case of Mexico, the economic sectors responded differently to this crisis, with the secondary and tertiary sectors being the hardest hit.

## Empirical evidence of uncertainty in the productive sectors

Table 1 presents the results of the Augmented Dickey–Fuller (ADF) tests performed on the data series under study. The Augmented Dickey–Fuller (ADF) test tests the null hypothesis that a unit root is present in a sample of time series. In all cases, the number of lags was determined according to Schwarz’s informative criterion, allowing a maximum of 4 lags. In the case of the natural logarithms of the international price of oil and of the sectoral products, it is not possible to reject the null hypothesis of unit root, with the exception of the natural logarithm of agricultural GDP when the test is specified with intercept and trend. On the contrary, in the case of the annual growth rates of all variables in all cases, the null hypothesis of unit root is rejected. From these results, it is possible to conclude that the series has a unit root in their levels, but their annualized growth rates are stationary.

**Table 1:** Augmented Dickey–Fuller (ADF) tests

*Variable*	A			B			C		
*Log of real oil price in pesos*	0.2490		(0)	−2.1543		(0)	−1.6296		(0)
*Log of real oil price in dollars*	−1.1347		(0)	−1.7190		(0)	−1.6079		(0)
*Log of agricultural GDP*	2.5344		(1)	−0.1752		(1)	−**3.9541**	**	(1)
*Log of industrial GDP*	0.8272		(0)	−2.2690		(0)	−2.9451		(0)
*Log of services GDP*	3.0119		(0)	−1.1599		(0)	−2.9326		(0)
*Oil growth*	−**9.2035**	***	(0)	−**9.2825**	***	(0)	−**9.4178**	***	(0)
*Agricultural GDP growth*	−**21.1473**	***	(0)	−**21.8160**	***	(0)	−**21.7414**	***	(0)
*Industrial GDP growth*	−**12.5924**	***	(0)	−**12.6332**	***	(0)	−**12.6958**	***	(0)
*Services GDP growth*	−**10.3286**	***	(0)	−**11.1700**	***	(0)	−**11.1826**	***	(0)

Source: authors using data from INEGI

Test statistics in bold indicate a rejection of the null hypothesis. The numbers in parentheses correspond to the number of lags in the test. The critical values at the significance level for Augmented Dickey–Fuller are −1.94, without constant and trend (model A), −2.86, including constant but no trend (model B), and −3.41, including constant and trend (model C)

**Significance of the test at 5%

***Significance of the test at 1%

Table 2 presents the results of the Kwiatkowski–Phillips–Schmidt–Shin KPSS (Kwiatkowski et al. 1992) applied to our data. These tests are used to test a null hypothesis that an observable time series is stationary around a deterministic trend (i.e., trend-stationary) against the alternative of a unit root. The presence of a unit root is not the null hypothesis but the alternative. For most of the variables in natural logarithms, the null hypothesis of stationarity is rejected, with the exception of the GDP of the services sector. Conversely, for the annual growth rates of the variables, in no case is it possible to reject the null hypothesis of stationarity. These results reinforce those obtained with the ADF unit root test, that the natural logarithms of the series are integrated of order 1 and stationary in their growth rates.

**Table 2:** Kwiatkowski, Phillips, Schmidt, y Shin (KPSS) tests

*Variable*	KPSS					
	*η*_*μ*_			*η*_*τ*_		
*Log of real oil price in pesos*	**1.0233**	***	(9)	**0.2919**	***	(8)
*Log of real oil price in dollars*	**0.7196**	**	(9)	**0.2425**	***	(8)
*Log of agricultural GDP*	**1.1966**	***	(9)	**0.1274**	*	(7)
*Log of industrial GDP*	**0.9872**	***	(9)	**0.2254**	***	(8)
*Log of services GDP*	**1.1943**	***	(9)	0.0816		(7)
*Oil growth*	0.2607		(0)	0.0272		(4)
*Agricultural GDP growth*	0.0481		(4)	0.0292		(4)
*Industrial GDP growth*	0.1528		(4)	0.0277		(4)
*Services GDP growth*	0.1106		(4)	0.0500		(4)

Source: authors using data from INEGI

The number of lags in each test is the number in parentheses; the test statistics in bold indicate the rejection of the null hypothesis. *ημ* and *η*_*τ*_ represent the statistics of the test where the null hypothesis poses that the series is stationary in level or around a deterministic trend, respectively

*Significance of the test at 10%

**Significance of the test at 5%

***Significance of the test at 1%

In addition, Appendix Table 7 presents the results of the Lee and Strazicich (2003) unit root tests with a mean structural break (model A) applied to the logarithms of real international oil prices, in pesos and in dollars, and to the GDP of each sector of economic activity in Mexico, as well as the annualized growth rates of these variables.

As can be seen in Appendix Table 7, it is not possible to reject the null hypothesis of unit root for the natural logarithm of all the variables, with the exception of the primary sector GDP, in which the break is not statistically significant in the test at the five percent significance level. On the contrary, in the case of the tests carried out on the growth rates of both the oil price and the sector’s GDP in Mexico, the null hypothesis of the unit root was rejected in all cases.

Similar results were found for the tests with two ruptures in Appendix Table 8. The results with one and two structural ruptures corresponding to model C, which can be found in Appendix Table 9, tend to confirm the previous results; the series are integrated of the first order in their levels logarithms and stationary in their annualized growth rates.

Taking into account that the series considered for the analysis are stationary, we estimate different bivariate VAR models with GARCH in mean effects for each sector’s GDP growth and for the growth of the real international price of oil, in dollars and in pesos, as described in Eqs. (1) and (3), by using the maximum likelihood method. Be reminded that the main goal is to determine if the volatility of the oil price affects real product growth for each economic sector. Based on what was proposed by Hamilton (1996), Hamilton and Herrera (2004), Edelstein and Kilian (2007), and Elder and Serletis (2009), the SVAR was specified with four lags, equivalent to one year with quarterly data, under the argument that oil price shocks affect real economic activity with a one-year delay.

There are other reasons behind this choice; for example, Hamilton and Herrera (2004) argue that a shorter period could conceal the response of economic activity to the oil shock, given that its influence may appear with a delay. In addition, it is argued that a greater number of lags are necessary for robustness in the results of nonlinear VAR models. Finally, this number of lags allows comparisons with other empirical studies carried out on the subject, since most of them use the number of lags equivalent to one year (Elder and Serletis 2009, 2010, 2011; Rahman and Serletis 2012; Aye et al. 2014). In this way, the effective sample for the two models estimated comprises the period from the second quarter of 1994 to the last quarter of 2020. The informative Schwarz criterion for both the homoskedastic VAR and the SVAR-MGARCH in the mean for each of the estimated models will determine whether the specification is consistent with the data. This criterion penalizes to a greater extent the inclusion of additional parameters, which in this case are required to estimate the GARCH model, so a lower criterion for the model with GARCH in mean effects is regarded as strong evidence in favor of the latter specification. Table 3 presents the results of estimating this criterion for both models.

**Table 3:** Model specification tests, 1993:2–2020:4

*VAR model and sample*	VAR	VAR with MGARCH-M
Price of oil in real dollars		
*Real oil price and agricultural GDP*	2.123	2.113
*Real oil price and industrial GDP*	2.138	2.020
*Real oil price and services GDP*	2.015	1.899
Price of oil in real pesos		
*Real oil price and agricultural GDP*	2.105	2.103
*Real oil price and industrial GDP*	2.137	1.997
*Real oil price and services GDP*	2.021	1.890

Source: authors using data from INEGI

The values of the Schwarz criterion reported in Table 3 indicate that in all models, the bivariate VAR with GARCH in mean effects is better to capture the characteristics of the data compared to the conventional homoskedastic VAR. Table 4 presents the estimated coefficients of the mean variance function of the MGARCH model, which provides additional support for both specifications. Table 4 also displays the impact of real oil price uncertainty on economic growth by sectors in Mexico, measured through the coefficient *H*_1, 1_(*t*)^1/2^, with its respective asymptotic *t*. This parameter represents the conditional volatility of changes in real oil prices in the equation of average growth of each sector’s real production. We find that the coefficients *H*_1, 1_(*t*)^1/2^ were negative and statistically significant for the secondary and tertiary sectors when the international price of oil was measured in dollars, but the coefficient of the primary sector was not statistically significant, in spite of being negative. Regarding the magnitude of the coefficients mentioned, the largest in absolute value was that of the secondary sector, which suggests that the greatest impact of international oil price uncertainty occurs in the secondary sector, followed by the tertiary sector according to this indicator.

**Table 4:** Estimated oil volatility coefficients

	Oil volatility coefficient
*Economic activity indicator*	*H*_1, 1_(*t*)^1/2^
Oil price in dollars	
*Agricultural GDP*	−0.008
	(0.22)
*Industrial GDP*	−0.173**
	(22.63)
*Services GDP*	−0.007**
	(2.10)
Oil price in pesos	
*Agricultural GDP*	0.016
	(0.21)
*Industrial GDP*	−0.151**
	(1134.93)
*Services GDP*	0.009**
	(45.22)

Source: authors using data from INEGI

The numbers in parentheses are the absolute values of asymptotic *t*-statistics

*Significance at the 10% level

**Significance at the 5% level

When the model includes the real international price of oil in pesos, the coefficients that capture the impact of uncertainty in economic growth by sectors were also statistically significant in the secondary and tertiary sectors, but it only had the expected sign in the secondary sector, thus suggesting that the exchange rate plays an important role in the impact of uncertainty on sectoral economic growth in Mexico, given that, in this case, it seems to slightly stimulate growth in the tertiary sector.

In this way, unforeseen shocks to the international price of oil, whether positive or negative, tend to increase the conditional standard deviation of oil. This increase has no impact on the growth of the primary sector; it will reduce the growth of the secondary sector, and the effect on the tertiary sector is ambiguous in Mexico in the short term. Such results are consistent with those reported by Elder and Serletis (2010, 2011), Rahman and Serletis (2012), Bredin et al. (2011), and Aye et al. (2014), who find that oil price volatility negatively influences the aggregate economic activity of Canada, the USA, South Africa, and the G-7 countries.

Table 5 presents the results of the ARCH effects tests on the residuals of the estimated equations. In no case is it possible to reject the null hypothesis of no ARCH effects in the residuals of the equations for each of the sectors, both when the price of oil is expressed in dollars and in pesos. This allows us to obtain valid inferences from the estimates made.

**Table 5:** ARCH effects tests on the residuals of the estimated equations

	*Agricultural GDP*		*Industrial GDP*		*Services GDP*	
	*u*_1_	*u*_2_	*u*_1_	*u*_2_	*u*_1_	*u*_2_
Oil price in dollars						
*Statistic*	1.432	1.935	0.505	1.935	0.395	0.513
*Signif. level*	[0.2293]	[0.2811]	[0.7322]	[0.1106]	[0.8118]	[0.7260]
Oil price in pesos						
*Statistic*	1.708	1.201	0.551	1.812	0.523	0.485
*Signif. level*	[0.1544]	[0.3151]	[0.6984]	[0.1326]	[0.7189]	[0.7468]

Source: authors using data from INEGI

*u*_1_ corresponds to the equation for annualized oil price returns and *u*_2_ to the equation for economic growth

Table 6 shows the serial autocorrelation tests carried out through the Ljung–Box statistic with 15 lags. There is no serial autocorrelation in the residuals of the estimated VAR model, that is, in the equations of the mean.

**Table 6:** Serial correlation tests on the VAR model residuals**.**

	*Agricultural GDP*		*Industrial GDP*		*Services GDP*	
	*u*_1_	*u*_2_	*u*_1_	*u*_2_	*u*_1_	*u*_2_
Oil price in dollars						
*Ljung–Box Q-statistics (15)*	6.176	11.545	7.239	3.848	7.527	3.302
*Signif. level*	[0.9766]	[0.7131]	[0.9507]	[0.9982]	[0.8118]	[0.7260]
Oil price in pesos						
*Ljung–Box Q-statistics (15)*	9.058	11.207	4.652	2.287	6.697	2.672
*Signif. Level*	[0.8745]	[0.7378]	[0.9947]	[0.9999]	[0.9655]	[0.9998]

Source: authors using data from INEGI

*u*_1_ corresponds to the equation for annualized oil price returns and *u*_2_ to the equation for economic growth. The numbers in parentheses correspond to the number of lags in the test

The impulse-response functions obtained from the simulation of the parameters estimated from the maximum likelihood method will evaluate the degree of symmetry (asymmetry) in the response of real economic activity by sectors in Mexico to an uncertainty shock in the real international price of oil.

**Fig. 6:**
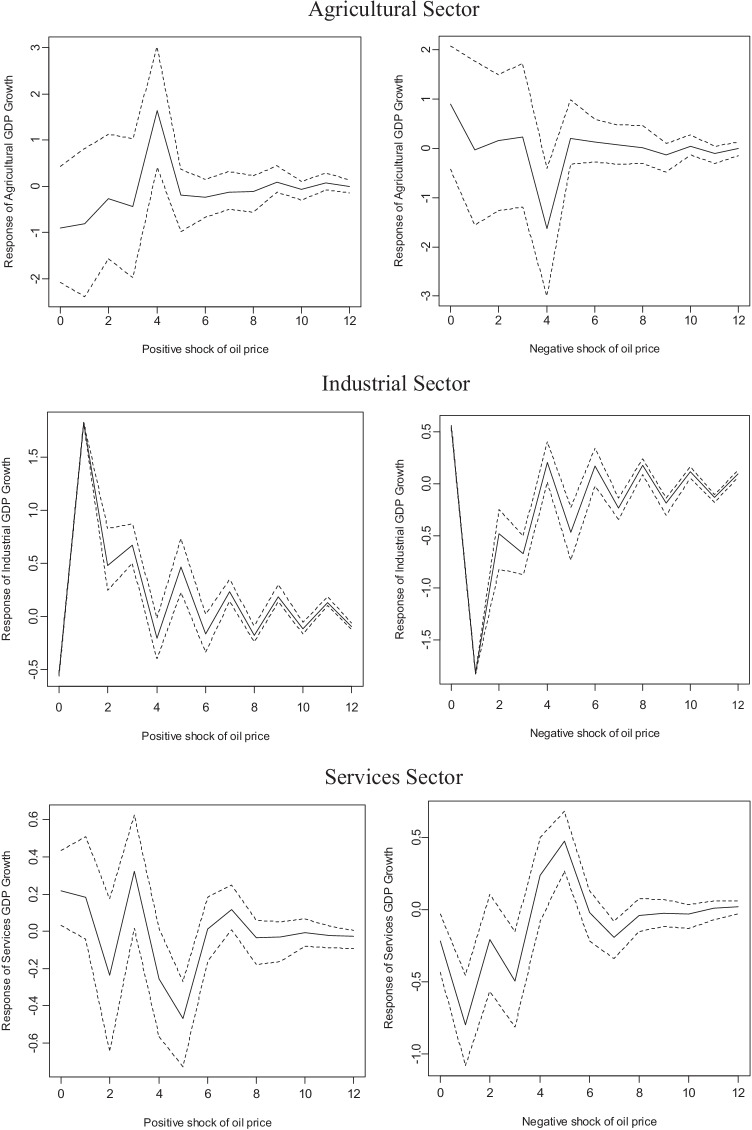
Impulse-response functions of one standard deviation to positive and negative shocks in the real price of oil in dollars. Source: authors using data from INEGI

Figure 6 shows these impulse-response functions over a 3-year horizon in economic growth by activity sectors in Mexico in the face of positive and negative shocks in the real price of oil. The magnitude of the shock equals the annualized unconditional standard deviation of changes in the price of oil. The confidence bands correspond to one standard error and are the dotted lines in Fig. 6, constructed from 1000 repetitions following the literature. The horizontal axis represents the forecast horizon, and the ordinate axis represents the responses of economic growth at the sector level to shocks in the real price of oil.

The results in the chart corroborate what was reported in Table 4, that positive and negative shocks to the international real price of oil do not have an impact on the economic growth of the primary sector, given that the impulse-response functions were not statistically significant. However, there is evidence of asymmetric effects on the growth of the secondary sector; in the face of positive shocks to the real price of oil in dollars, the impact was initially negative and later became positive, while in the face of negative shocks, the initial impact was positive and later became negative. In the tertiary sector, there was also evidence of asymmetric effects; a positive shock in the international real price of oil caused a positive impact on the economic growth of the tertiary sector in the short term, while a negative shock in the international real price of oil caused a negative effect.

**Fig. 7:**
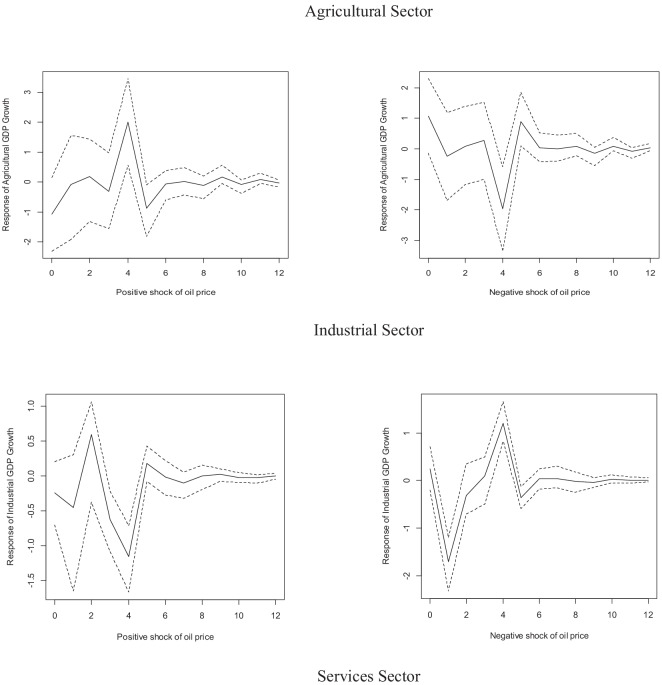
Impulse-response functions of one standard deviation to positive and negative shocks in the real price of oil in pesos. Source: authors using data from INEGI

Figure 7 presents the asymmetric impact of shocks to the real international price of oil expressed in local currency. In line with the results obtained for the real price of oil, there is no evidence that shocks to the real price of oil in pesos immediately impact the economic growth of the primary sector, with the exception of a negative impact after one year. Regarding the possible asymmetric impact of these shocks in the secondary sector, the effect of positive shocks in the second quarter after the shock is positive, but it turns to negative after the fourth quarter; meanwhile, negative shocks have a negative effect during the two first quarters after the shock and a positive effect after one year. The asymmetric impact of oil price shocks is more evident in the tertiary sector, given that positive shocks positively impact the growth of this sector, while negative shocks negatively impact said growth.

Thus, we find evidence that international oil price shocks in real terms have asymmetric effects on the economic growth of the different sectors that make up the economy of Mexico.

## Conclusions

This paper has shown that the uncertainty in the real price of oil affects real economic activity at the sectoral level in the Mexican economy, using quarterly data from 1993 to 2020. Mexico depends on oil sales to foster economic growth; the fact that there are international fluctuations in the price of this commodity negatively affects its growth and, at the same time, influences economic activities that constitute the sustenance of Mexicans through employment and wages, all of which makes this a relevant research topic.

Results from the bivariate structural VAR model, in which the uncertainty is endogenous and predetermined, indicate that oil price shocks have a negative and significant effect on the real economic activity of the secondary sector in Mexico, regardless of how the real price is measured, either in dollars or in constant pesos. The impact on the tertiary sector gathered mixed evidence, negative when the oil price was in dollars and positive when it was in pesos. There was no evidence of any effect on the primary sector. Additionally, the impulse-response functions demonstrated that oil price shocks have asymmetric effects in the secondary and tertiary sectors of economic activity in Mexico and confirmed that the effect on the primary sector is practically nonexistent. Results can be attributed to the fact that the use of oil as a production input may be larger in the economic activities of the secondary sector, such as manufacturing, while it is probably less used in the tertiary sector, characterized by a rather heterogeneous composition, ranging from activities such as financial services to health, education, and transportation, the latter of which is where oil price shocks may have a greater impact. The null impact of uncertainty about real oil prices in the primary sector is perhaps due to weaker ties to international trade so that the demand for the products generated there is inelastic to the price of oil.

The asymmetry in the results at the sectoral level of economic activity found in this paper shows that more research is required to address the impact of oil price shocks in emerging economies – such as Mexico – with a higher level of disaggregation of economic activities. This would enable the design and planning of economic policy measures and instruments that are better able to reduce the adverse effects of these shocks on the different economic activities. In future works, it could be analyzed how the international price of oil generated by the war between Russia and Ukraine has influenced the well-being of the population, impacting not only fuel prices, but now more resources have to be allocated to acquire those fuels, which are necessary to transport the goods and services that the population requires, thereby generating a generalized and sustained increase in prices in different countries, including Mexico, which is not analyzed in this work.

To finish, it is worth discussing potential implications for economic policy. Firstly, the need for energy policies that contribute to economic stability and reduce the adverse effect of oil price volatility in the secondary and tertiary sectors of economic activity becomes evident. These policies may include incentives for vehicles to optimize fuel consumption and for the industrial sector to improve the efficiency of its fuel or diesel power plants; such incentives could be directed to the sectors that make use of renewable and cleaner energy – an actual possibility in the Mexican economy enabled by recent law changes in this regard – and thus contribute to reducing the dependence on oil. It could also be advisable to promote tax cuts as an incentive for lowering energy consumption. In this way, the government must promote the use of clean energy by sponsoring investment and development in these areas and generate better conditions for companies to invest in activities that employ the use of renewable energy.

Among other measures that may be useful is the reduction of dependence on oil for public spending. The use of renewable energy is growing worldwide and will eventually displace sectors that continue to use fossil fuels. Emerging countries such as Mexico, which still continue to focus their efforts on oil, and depending economically on it as well, should start looking for alternatives so that their economies do not collapse in the future. Adapting to global needs in the use of renewable energy is not only a human responsibility but also an economic obligation. In this way, reducing the dependence on oil would also reduce the negative effects on Mexican economic sectors.

## Data Availability

All data generated or analyzed during this study are included in this article.

## Appendix

**Table 7:** Endogenous unit root test of the Lagrange multiplier with one structural break

*Log of real oil price*				
*Model A: k = 0, T*_*B1*_ *= 2014:3, N = 110*				
Parameter	*μ*_*0*_	*d*_*1*_	*d*_*2*_	*ϕ*
Coefficient	0.0252	−0.4268		−0.0737
*t*-statistic	1.4275	−2.6752		−2.0232
*Log of agricultural GDP*				
*Model A: k = 0, T*_*B1*_ *= 2008:3, N = 110*				
Parameter	*μ*_*0*_	*d*_*1*_	*d*_*2*_	*ϕ*
Coefficient	−0.0159	−0.0332		−0.8609
*t*-statistic	−4.2320	−1.0720		−8.9926
*Log of industrial GDP*				
*Model A: k = 0, T*_*B1*_ *= 1996:2, N = 110*				
Parameter	*μ*_*0*_	*d*_*1*_	*d*_*2*_	*ϕ*
Coefficient	0.0193	0.0254		−0.1951
*t*-statistic	3.2501	0.7162		−3.4008
*Log of services GDP*				
*Model A: k = 0, T*_*B1*_ *= 2017:1, N = 110*				
Parameter	*μ*_*0*_	*d*_*1*_	*d*_*2*_	*ϕ*
Coefficient	0.0127	0.0008		−0.1446
*t*-statistic	4.2194	0.0367		−2.8882
*Oil growth*				
*Model A: k = 0, T*_*B1*_ *= 1998:3, N = 110*				
Parameter	*μ*_*0*_	*d*_*1*_	*d*_*2*_	*ϕ*
Coefficient	−10.8970	1.0465		−0.8817
*t*-statistic	−1.7051	0.0159		−9.1853
*Agricultural GDP growth*				
*Model A: k = 0, T*_*B1*_ *= 2004:4, N = 110*				
Parameter	*μ*_*0*_	*d*_*1*_	*d*_*2*_	*ϕ*
Coefficient	12.1375	−23.9261		−1.6192
*t*-statistic	8.9061	−1.8355		−21.3294
*Industrial GDP growth*				
*Model A: k = 0, T*_*B1*_ *= 1996:2, N = 110*				
Parameter	*μ*_*0*_	*d*_*1*_	*d*_*2*_	*ϕ*
Coefficient	−10.5830	26.7239		−1.0765
*t*-statistic	−5.9836	1.7016		−11.1681
*Services GDP growth*				
*Model A: k = 0, T*_*B1*_ *= 1996:1, N = 110*				
Parameter	*μ*_*0*_	*d*_*1*_	*d*_*2*_	*ϕ*
Coefficient	−5.7186	7.8244		−0.9483
*t*-statistic	−5.4365	0.8547		−9.8222
Null hypothesis: *y*_*t*_ = *μ*_0_ + *d*_1_*B*_1*t*_ + *d*_2_*B*_2*t*_ + *y*_*t* − 1_ + *v*_1*t*_				
Alternative hypothesis: *y*_*t*_ = *μ*_1_ + *γt* + *d*_1_*D*_1*t*_ + *d*_2_*D*_2*t*_ + *v*_2*t*_				
where *D*_*jt*_ = 1 for *t* ≥ *T*_*Bj*_ + 1, *j* = 1, 2 and 0 otherwise; *B*_*jt*_ = 1 for *t* ≥ *T*_*Bj*_ + 1, *j* = 1, 2 and 0; otherwise, *T*_*Bj*_ is the break date				

Source: authors using data from INEGI

**Table 8:** Endogenous unit root test of the Lagrange multiplier with two structural breaks

*Log of real oil price*				
*Model A: k = 0, T*_*B1*_ *= 2005:1, T*_*B2*_ *= 2014:3, N = 110*				
Parameter	*μ*_*0*_	*d*_*1*_	*d*_*2*_	*ϕ*
Coefficient	0.0258	0.2068	−0.4254	−0.0877
*t*-statistic	1.4506	1.3014	−2.6803	−2.2047
*Log of agricultural GDP*				
*Model A: k = 0, T*_*B1*_ *= 2008:3, T*_*B2*_ *= 2008:3, N = 110*				
Parameter	*μ*_*0*_	*d*_*1*_	*d*_*2*_	*ϕ*
Coefficient	−0.0129	−0.0238	0.0153	−0.9801
*t*-statistic	−3.9017	−0.8112	0.5211	−10.0930
*Log of industrial GDP*				
*Model A: k = 0, T*_*B1*_ *= 1996:2, T*_*B2*_ *= 1999:3, N = 110*				
Parameter	*μ*_*0*_	*d*_*1*_	*d*_*2*_	*ϕ*
Coefficient	0.0195	0.0264	0.0324	−0.2165
*t*-statistic	3.3427	0.7495	0.9204	−3.5874
*Log of services GDP*				
*Model A: k = 0, T*_*B1*_ *= 1999:3, T*_*B2*_ *= 2017:1, N = 110*				
Parameter	*μ*_*0*_	*d*_*1*_	*d*_*2*_	*ϕ*
Coefficient	0.0123	0.0159	0.0015	−0.1506
*t*-statistic	4.2137	0.7678	0.0721	−2.9378
*Oil growth*				
*Model A: k = 0, T*_*B1*_ *= 1998:3, T*_*B2*_ *= 2007:3, N = 110*				
Parameter	*μ*_*0*_	*d*_*1*_	*d*_*2*_	*ϕ*
Coefficient	−11.9112	−4.2069	5.8962	−0.8956
*t*-statistic	−1.8521	−0.0639	0.0895	−9.2718
*Agricultural GDP growth*				
*Model A: k = 0, T*_*B1*_ *= 2004:4, T*_*B2*_ *= 2012:1, N = 110*				
Parameter	*μ*_*0*_	*d*_*1*_	*d*_*2*_	*ϕ*
Coefficient	13.6086	−22.2218	8.2183	−1.6295
*t*-statistic	9.8208	−1.7215	0.6399	−21.5910
*Industrial GDP growth*				
*Model A: k = 0, T*_*B1*_ *= 1996:2, T*_*B2*_ *= 1994:4, N = 110*				
Parameter	*μ*_*0*_	*d*_*1*_	*d*_*2*_	*ϕ*
Coefficient	−9.4255	25.0439	3.7141	−1.0959
*t*-statistic	−5.5156	1.6059	0.2388	−11.3349
*Services GDP growth*				
*Model A: k = 0, T*_*B1*_ *= 1996:1, T*_*B2*_ *= 2007:3, N = 110*				
Parameter	*μ*_*0*_	*d*_*1*_	*d*_*2*_	*ϕ*
Coefficient	−5.8926	7.6491	−5.0318	−0.9702
*t*-statistic	−5.5772	0.8408	−0.5540	−9.9937
Null hypothesis: *y*_*t*_ = *μ*_0_ + *d*_1_*B*_1*t*_ + *d*_2_*B*_2*t*_ + *y*_*t* − 1_ + *v*_1*t*_				
Alternative hypothesis: *y*_*t*_ = *μ*_1_ + *γt* + *d*_1_*D*_1*t*_ + *d*_2_*D*_2*t*_ + *v*_2*t*_				
where *D*_*jt*_ = 1 for *t* ≥ *T*_*Bj*_ + 1, *j* = 1, 2 and 0 otherwise; *B*_*jt*_ = 1 for *t* ≥ *T*_*Bj*_ + 1, *j* = 1, 2 and 0; otherwise, *T*_*Bj*_ marks the break date.				

Source: authors using data from INEGI

**Table 9:** Endogenous unit root test of the Lagrange multiplier with two structural breaks (model C)

*Variable*	${\hat{T}}_B$	*statistic*	${\hat{D}}_{1t}$	$\hat{d}{t}_1^{\ast }$	${\hat{D}}_{2t}$	$\hat{d}{t}_2^{\ast }$
*Log of real oil price*	2011:1	−3.3314	−0.0463	0.0118		
			−0.2890	0.3217		
	2003:4,2014:3	−4.0936	−0.0373	0.1606	−0.4475	−0.0983
			−0.2398	3.1828	−2.8817	−2.2598
*Log of agricultural GDP*	2010:2	−10.1971	−0.0556	0.0113		
			−1.8918	1.9367		
	2006:3,2011:3	−11.2407	0.0184	0.0159	0.0216	0.0149
			0.6476	2.0710	0.7658	1.8750
*Log of industrial GDP*	1996:04	−4.3733	0.0021	0.0264		
			0.0612	2.2308		
	1997:2, 2017:4	−5.1792	0.0117	0.0164	0.0180	−0.0272
			0.3493	1.6567	0.5133	−2.3310
*Log of services GDP*	2017:03	−4.0996	0.0153	−0.0081		
			0.7425	−1.2779		
	2008:2, 2017:4	−4.7215	−0.0204	−0.0042	0.0077	−0.0061
			−1.0408	−1.0233	0.3825	−0.8666
*Oil growth*	2014:1	−9.1660	−24.8665	−5.8030		
			−0.3705	−0.3866		
	1998:3,2001:2	−9.5087	−49.4585	67.4401	160.7751	−85.0177
			−0.7320	2.6980	2.4871	−3.6993
*Agricultural GDP growth*	2010:2	−21.6815	−41.9521	19.8754		
			−3.2408	7.3968		
	2000:1,2004:4	−22.0130	−43.0617	21.7216	−16.9007	−12.6792
			−3.3008	5.5287	−1.3262	−3.7041
*Industrial GDP growth*	1996:2	−12.6746	27.0355	−16.5103		
			1.8463	−3.6958		
	1997:4,2017:4	−12.7793	−1.7198	−3.3586	9.5431	−10.1030
			−0.1179	−0.9053	0.6264	−2.0419
*Services GDP growth*	2017:3	−11.1712	10.7510	−9.8929		
			1.1970	−3.4405		
	2005:1,2009:1	−11.4899	1.6236	0.6267	32.9111	−13.5028
			0.1885	0.2538	3.8722	−4.8639
